# Nitrogen Regulating the Expression and Localization of Four Glutamine Synthetase Isoforms in Wheat (*Triticum aestivum* L.)

**DOI:** 10.3390/ijms21176299

**Published:** 2020-08-31

**Authors:** Yihao Wei, Xiaochun Wang, Zhiyong Zhang, Shuping Xiong, Xiaodan Meng, Jie Zhang, Lulu Wang, Xiaojiao Zhang, Meiqin Yu, Xinming Ma

**Affiliations:** 1 Collaborative Innovation Center of Henan Grain Crops, College of Agronomy, Henan Agricultural University, Zhengzhou 450000, China; yihaowei@stu.henau.edu.cn (Y.W.); zhiyongzhang@henau.edu.cn (Z.Z.); shupxiong@henau.edu.cn (S.X.); xiaodanmeng@stu.henau.edu.cn (X.M.); zhangjie135239@stu.henau.edu.cn (J.Z.); wll9501@stu.henau.edu.cn (L.W.); huawu@stu.henau.edu.cn (X.Z.); 2 Department of Biochemistry and Molecular Biology, College of Life Science, Henan Agricultural University, Zhengzhou 450000, China; yumeiqin@stu.henau.edu.cn

**Keywords:** glutamine synthetase, protein level, kinetic property, localization, nitrogen, wheat

## Abstract

Glutamine synthetase (GS), the key enzyme in plant nitrogen assimilation, is strictly regulated at multiple levels, but the most relevant reports focus on the mRNA level. Using specific antibodies as probes, the effects of nitrogen on the expression and localization of individual wheat GS (TaGS) isoforms were studied. In addition to TaGS2, TaGS1;1 with high affinity to substrate and TaGS1;3 with high catalytic activity were also localized in mesophyll, and may participate in cytoplasmic assimilation of ammonium (NH_4_^+^) released from photorespiration or absorbed by roots; TaGS1;2 was localized in xylem of leaves. In roots, although there were hundreds of times more *TaGS1;1* than *TaGS1;2* transcripts, the amount of TaGS1;1 subunit was not higher than that of TaGS1;2; NH_4_^+^ inhibited TaGS1;1 expression but stimulated TaGS1;3 expression. In root tips, nitrate stimulated TaGS1;1, TaGS1;3, and TaGS2 expression in meristem, while NH_4_^+^ promoted tissue differentiation and TaGS1;2 expression in endodermis and vascular tissue. Only TaGS1;2 was located in vascular tissue of leaves and roots, and was activated by glutamine, suggesting a role in nitrogen transport. TaGS1;3 was induced by NH_4_^+^ in root endodermis and mesophyll, suggesting a function in relieving NH_4_^+^ toxicity. Thus, TaGS isoforms play distinct roles in nitrogen assimilation for their different kinetic properties, tissue locations, and response to nitrogen regimes.

## 1. Introduction

Nitrogen (N) is a key limiting factor in the yield and quality of crops, and large quantities of nitrogen fertilizers are required to attain maximal growth and productivity [1,2]. To increase crop production, nitrogen fertilizers are often applied excessively, leading to severe nitrogen pollution on a global scale [3,4]. Therefore, there is a need to improve nitrogen use efficiency (NUE) to make agriculture more sustainable [4,5].

In order to improve crop NUE, glutamine synthetase (GS; EC 6.3.1.2) has been studied numerous times owing to its essential role in the assimilation of inorganic N [1,6,7,8,9,10]. GS catalyzes the ATP-dependent fixation of ammonium (NH_4_^+^) to glutamate (Glu) to form glutamine (Gln) [11]. Plant GS is classified into two groups according to its subcellular location: Cytosolic glutamine synthetase (GS1) and chloroplast glutamine synthetase (GS2) [12,13]. GS2 is encoded by a single gene, while GS1 is encoded by a multigene family [5].

Although all GS isozymes are involved in Gln synthesis, GS isozymes play different roles in nitrogen assimilation or transportation in plants. GS2 is involved in assimilating NH_4_^+^ derived from photorespiration and nitrate (NO_3_^−^) reduction [14,15]. GS1 has multiple isoforms with distinct affinities for NH_4_^+^ and glutamate [16,17], and each GS1 isoform may have a different function in nitrogen assimilation or transportation. Wheat is an important crop for mankind. individual wheat GS (TaGS) isozymes are classified into four subfamilies: TaGS1, TaGSr, TaGSe, and TaGS2 [9]. Thomsen et al. clustered GS isozymes of cereals into four categories: GS1;1, GS1;2, GS1;3, and GS2 [5]. Based on the cluster of TaGS isoforms, we renamed TaGS1, TaGSr, and TaGSe genes as TaGS1;1, TaGS1;2, and TaGS1;3, respectively.

The physiological functions of GS isozymes have been studied according to the cellular localization and expression characteristics. In Arabidopsis, the green fluorescent protein (GFP) signal driven by the *AtGln1;1* promoter is recorded in the epidermal cells of the root elongation zone and can affect primary root development in response to exogenous N provision [18]. The promoter of *AtGln1;2* can drive reporter gene expression in the mesophyll and vasculature of developed leaves [19,20]; vascular cells, cortex, and epidermis of roots [18,21]; epidermal cells of sepals; and veins of petals and stamens [18]. The mRNA level of *AtGln1;2* can be upregulated to relieve NH_4_^+^ toxicity under ample nitrate (NO_3_^−^) supply and high NH_4_^+^ supply conditions [19,20,21]. Promoter::GFP fusion has shown that *AtGln1;3* expression is localized in the pericycle, suggesting a role in loading glutamine to the xylem [21]. A more recent study showed that β-glucuronidase (GUS) activity driven by *Gln1;1−5* promoters was localized in phloem companion cells but in veins of different order, and *AtGln1;1*, *AtGln1;2*, and *AtGln1;3* act together for N remobilization and seed filling [22].

In maize, *ZmGln1−3* in the mesophyll cells has a role in the synthesis of Gln following NO_3_^−^ reduction until plant maturity [7,23]. *ZmGln1−4* in bundle sheath cells has a role in the reassimilation of NH_4_^+^ released during protein degradation in senescing leaves [7,24]. In rice, *OsGS1;1*, with its transcript located in vascular tissue of mature leaves, has a role in grain filling [25,26]. *OsGS1;2*, with its transcript located in surface cells of roots in an NH_4_^+^-dependent manner, is important in the primary assimilation of NH_4_^+^ taken up by rice roots [16,27]. *OsGS1;3* transcript is mainly expressed in the spikelet, indicating a key role in grain ripening and/or germination [28]. In wheat, *TaGS1* transcript is present in the perifascicular sheath cells, and *TaGSr* transcripts are confined to the vascular cells [9,29]. During leaf senescence, *TaGS1* and *TaGSr* have high mRNA levels, suggesting major roles in assimilating ammonia during the critical phases of remobilization of nitrogen to the grain [9]. However, since GS genes are highly homologous and their gene products are indistinguishable at the protein level by any GS antibody, previous studies about the cellular localization and expression characteristics of individual GS isozymes were mainly focused on the mRNA level [9,12,29,30]. 

In cells, the inorganic nitrogen assimilation process that GS participates in consumes a substantial amount of energy; therefore, GS must be tightly regulated at the gene, transcript, and protein level [5,11,31,32]. The regulation of each step of this process may affect the localization and activity of GS. In transgenic alfalfa, constitutively overexpressed GS1 genes significantly increased the level of GS1 transcripts in the leaves, but it did not significantly change the level of GS1 polypeptides [33]. In our previous study, although there were numerous *TaGS2* transcripts in the roots of *TaGS2* overexpressed tobacco, TaGS2 proteins could not be detected [32]. These results showed that GS expression at the mRNA level could not completely reflect the content of GS protein. Therefore, a study on the expression pattern and localization of GS isozymes at the protein level will be more conducive to confirming and supplementing our knowledge of their functions.

To further study their functions at the protein level, specific antibodies of individual TaGS isoforms were prepared in our previous study, which could recognize specific individual TaGS subunits (Figure S1). By investigating the effects of nitrogen nutrition on the expression and localization of individual TaGS isoforms with specific antibodies as probes and determining their kinetic properties, some new functions of TaGS isoforms are proposed in this paper.

## 2. Results

### 2.1. Effects of Nitrogen on Expression of TaGS Isozymes at mRNA and Protein Level

GS proteins are responsible for catalytic activity; therefore, only when the mRNA level of TaGS is consistent with the relevant protein level can the mRNA level of TaGS represent its function to some degree. Here, the mRNA level of individual TaGS isoforms was determined by real-time PCR, and the protein level was analyzed by Western blot using specific antibodies of individual TaGS isoforms. 

**Figure 1:**
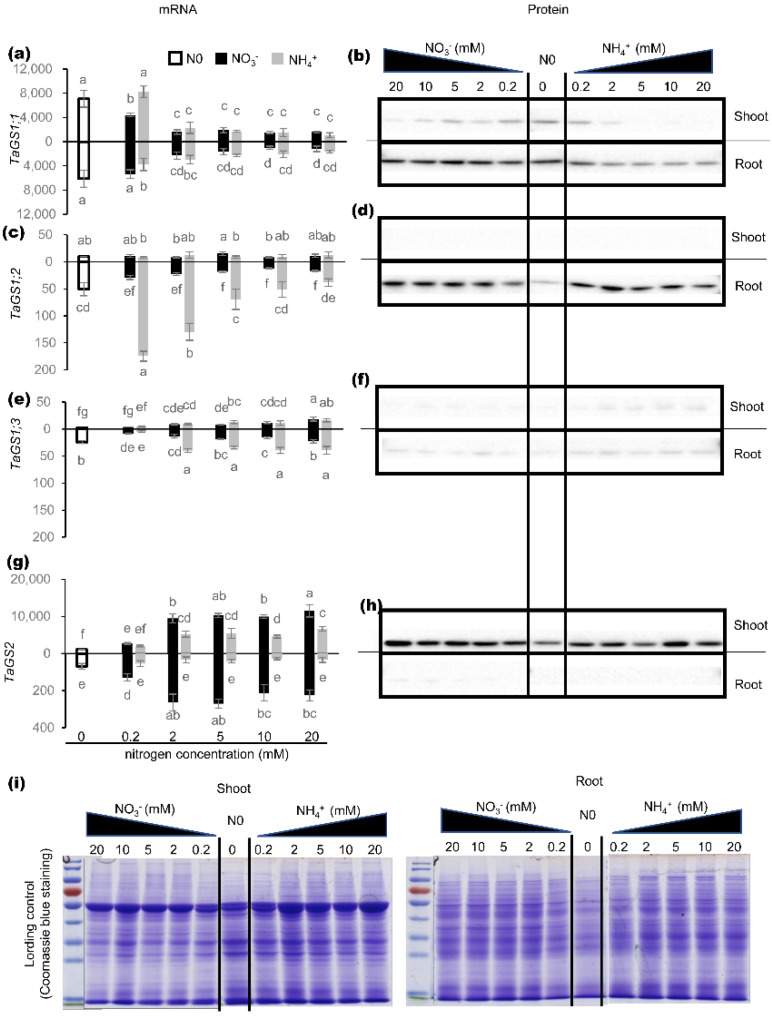
Expression patterns of individual individual wheat glutamine synthetase (TaGS) isoforms at mRNA and protein level. Quantitative RT-PCR analysis of (**a**) TaGS1;1, (**c**) TaGS1;2, (**e**) TaGS1;3, and (**g**) TaGS2 gene expression in response to different nitrogen regimes in shoots and roots. Horizontal axes show millimolar concentration of NO_3_^−^ and NH_4_^+^ treatments, vertical axes show mean relative expression of each isoform normalized to reference genes *TaATPase* and *TaTEF*. Data are means of three independent biological replicates ± SD. Letters above samples indicate statistically significant differences where *p* < 0.05 according to one-way ANOVA Duncan post-hoc test. Western-blot analysis of (**b**) TaGS1;1, (**d**) TaGS1;2, (**f**) TaGS1;3, and (**h**) TaGS2 protein content in response to different nitrogen regimes in shoots and root tissue. (**i**) Coomassie blue staining indicates equal total protein loading.

Different nitrogen treatments had distinct effects on both mRNA and protein of TaGS isoforms. Under different nitrogen levels, the amount of TaGS1;1 subunit in roots was significantly higher than that in shoots, which was not consistent with the results at the mRNA level (Figure 1a,b). Under 2–20 mM N supply, *TaGS1;1* transcript showed similar levels under NO_3_^−^ and NH_4_^+^ supply (Figure 1a), but the TaGS1;1 subunit showed a higher amount under NO_3_^−^ supply (Figure 1b). This inconsistency between mRNA and protein of TaGS1;1 may suggest that NO_3_^−^ can facilitate translation of TaGS1;1.

In roots, the transcription level of *TaGS1;2* was far lower than that of *TaGS1;1*, and decreased gradually with increased NH_4_^+^ concentration, but the content of TaGS1;2 subunit was the highest and remained basically unchanged (Figure 1c,d). In addition, the level of *TaGS1;2* transcripts under NH_4_^+^ supply was significantly higher than that under NO_3_^−^ supply (Figure 1c), but there was no significant difference in TaGS1;2 subunit content (Figure 1d). These results suggest that the translation of *TaGS1;2* transcript in roots was inhibited by NH_4_^+^.

Although *TaGS1;3* showed similar mRNA levels under NO_3_^−^ and NH_4_^+^ supply in shoots, the *TaGS1;3* transcript showed higher expression under NH_4_^+^ supply in roots (Figure 1e), suggesting that NH_4_^+^ can promote the expression of *TaGS1;3* in roots. In any situation, the amounts of TaGS1;3 subunit from western blot were hardly visible (Figure 1f).

TaGS2 showed similar expression patterns at the mRNA and protein level under different nitrogen treatments (Figure 1g,h). In roots, the level of *TaGS2* transcripts under NO_3_^−^ supply was significantly higher than that under NH_4_^+^ supply (Figure 1g), indicating that *TaGS2* in the root was induced by NO_3_^−^.

These results show that except for TaGS2, the expression patterns of TaGS1 isoforms at the mRNA level were not consistent with those at the protein level, and the expression characteristics and cellular localization of TaGS isoform at the protein level may really reflect its function.

### 2.2. Effects of Nitrogen on Cellular Localization of Individual TaGS Isozymes at Protein Level

Responses of cellular localization of TaGS to N nutrition are crucial to understand the role of individual TaGS isoforms in N metabolism. In order to determine the cellular localization of individual TaGS isoforms at the protein level, immunofluorescence analysis was carried out using specific antibodies of individual TaGS isoforms as probes. 

First, the histological structures of the leaves and the maturation and meristematic zones of roots under different nitrogen regimes were observed, and the results showed that 5 mM NH_4_^+^ treatment significantly changed the histological structure of the root tip meristematic zone (Figure S2).

**Figure 2:**
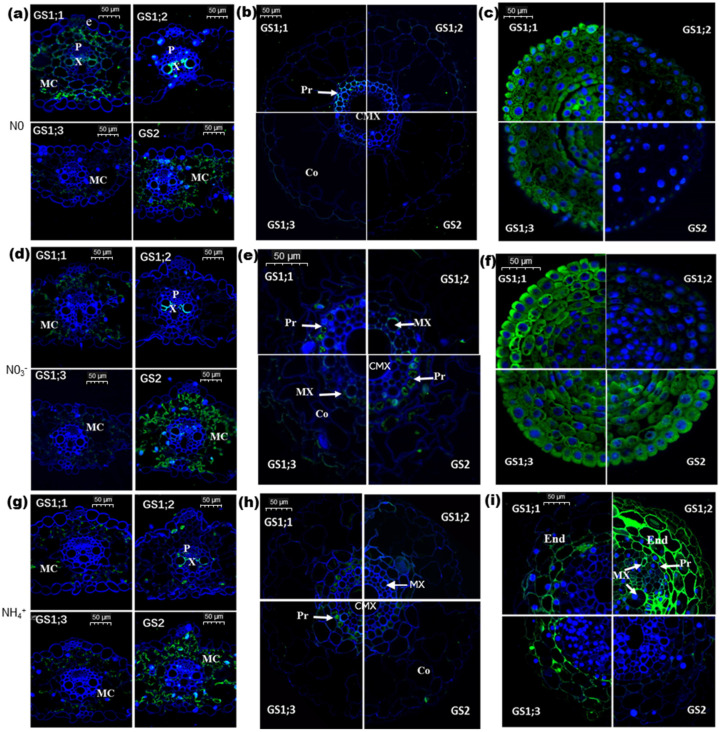
Cellular localization of individual TaGS at protein level. Three-day-old seedlings were separated and grown on a modified Hoagland nutrient solution for 12 days, without N supply (N0), or with 5 mM NO_3_^−^ or 5 mM NH_4_^+^ as the sole N source, and then the top fully expanded leaf, root, and root tips were prepared for immunolocalization. Immunolocalization of TaGS1;1, TaGS1;2, TaGS1;3, and TaGS2 in response to different nitrogen regimes in (**a**,**d**,**g**) a transverse section of the leaf, and (**b**,**e**,**h**) the maturation zone and (**c**,**f**,**i**) meristematic zone of root tissue. Visually, 4′,6-diamidino-2-phenylindole (DAPI) glowed blue by 330–380 nm UV excitation wavelength and 420 nm emission wavelength; fluorescein isothiocyanate (FITC) glowed green by 465–495 nm excitation wavelength and 515–555 nm emission wavelength. e, epidermis; MX, metaxylem; P, phloem; X, xylem; CMX, central metaxylem; End, endodermis; Pr, pericycle; Co, cortex; and MC, mesophyll cell.

Without N supply (N0), TaGS2 and TaGS1;1 were localized in leaf mesophyll cells and TaGS1;1 was also localized in the surrounding vessels of xylem in leaf veins (Figure 2a). TaGS1;2 was mainly localized in the surrounding vessels of xylem, while no obvious TaGS1;3 was detected in the leaf (Figure 2a). Only TaGS1;1 was detected in vascular bundles in the maturation zone of roots (Figure 2b), but abundant TaGS1;1 and TaGS1;3 was found in the meristematic zone of root tips (Figure 2c).

Under 5 mM NO_3_^−^ supply, the localization of TaGS in the leaves was very similar to that with N0 supply, but there were more TaGS2 and less TaGS1;1 in the mesophyll cells, and no TaGS1;1 was detected in the surrounding vessels of xylem (Figure 2d). In the maturation zone of roots, TaGS1;1 and TaGS2 were localized in pericycle cells, and TaGS1;2 and TaGS1;3 were localized in surrounding vessels of xylem (Figure 2e). Moreover, abundant TaGS1;1, TaGS1;3, and TaGS2 was detected in the meristematic zone of root tips (Figure 2f).

Under 5 mM NH_4_^+^ supply, the tissue localization of TaGS1;1, TaGS1;3, and TaGS2 in the leaves was the same as with 5 mM NO_3_^−^ supply. TaGS1;2 was localized in the surrounding vessels of xylem and phloem companion cells in the leaves (Figure 2g). In the maturation zone of roots, TaGS1;1 and TaGS1;3 were localized in the pericycle cells, and TaGS1;2 and TaGS2 were not detected (Figure 2h). In the same site of root tips, the supposed root tip meristem under NH_4_^+^ treatment was full of vascular tissue (Figure 2i, Figure S3). There was abundant TaGS1;2, TaGS1;3, and TaGS1;1 in the endodermis, but no TaGS2 was detected (Figure 2i). Moreover, a large amount of TaGS1;2 was detected in the surrounding vessels of xylem in the vascular bundle (Figure 2i). 

Under different N nutrition conditions, the cellular localization of individual TaGS proteins in the leaves and roots was different, suggesting that individual TaGS isoforms have different functions in N metabolism processes.

### 2.3. Effects of Nitrogen Nutrition on GS Isozyme and Total GS Activity

**Figure 3:**
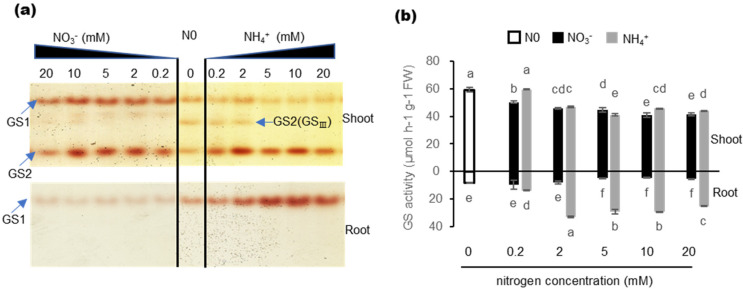
Glutamine synthetase (GS) activity in response to different nitrogen regimes in shoot and root tissue. (**a**) Native electrophoresis and in-gel GS activity staining showing the GS holoenzymes in shoot and root; (**b**) total GS activity in shoot and root tissue under different N regimes. Data are means of three independent biological replicates ± SD. Letters above samples indicate statistically significant differences where *p* < 0.05 according to one-way ANOVA Duncan post-hoc test.

To analyze the functions of TaGS isozymes under different N nutrition conditions, GS isozyme activity and total GS activity were determined. In previous studies, the cytosolic GS1 holoenzyme was ~490 kDa and the chloroplastic GS2 holoenzyme was ~240 kDa [34]. Therefore, the isoforms showed different mobility in gels (GS2 > GS1). In the shoot, the activity of both GS1 and GS2 holoenzyme could be detected, and the GS2 (GS_Ⅲ_) holoenzyme found by Wang et al. [34] was also found under the condition of N0 and 0.2–2 mM NH_4_^+^ supply (Figure 3a). The total GS activity in the shoot was significantly higher than that in the root (Figure 3b). In the root, only GS1 holoenzyme activity could be detected, and both GS1 holoenzyme activity and total GS activity under NH_4_^+^ supply were significantly higher than that under NO_3_^−^ supply (Figure 3a,b). However, the subunits of TaGS1;1 and TaGS1;2 did not show higher expression under NH_4_^+^ supply (Figure 1b,d). These results show that the activity of GS was not directly proportional to the content of GS subunit, but may be related to its catalytic properties. 

### 2.4. Kinetic Properties of Recombinant TaGS Isozymes

**Figure 4:**
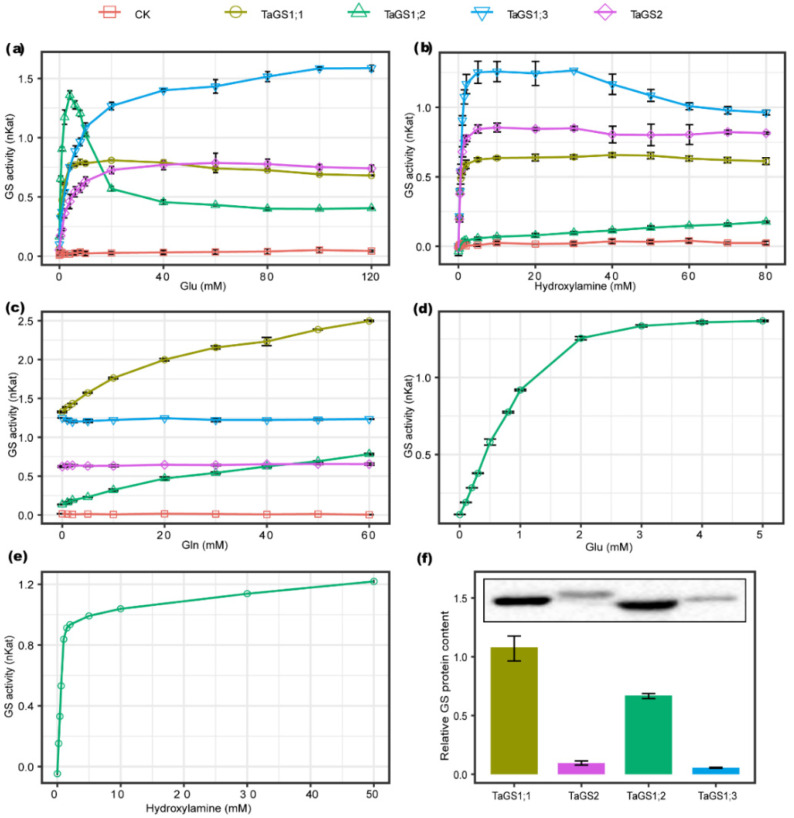
Kinetic properties of recombinant TaGS isozymes. Activity of individual recombinant wheat GS isozymes in relation to additional (**a**) glutamate (Glu), (**b**) hydroxylamine, and (**c**) glutamine (Gln). (**d**) TaGS1;2 activity was measured when Glu was supplied at concentrations of 0–5 mM. (**e**) TaGS1;2 activity was measured at different concentrations of hydroxylamine when Glu was supplied at 5 mM. Volume of individual recombinant wheat GS isozymes crude extract with 200 μL of TaGS1;1, 150 μL of TaGS1;2, 450 μL of TaGS1;3, and 300 μL TaGS2 was used for GS enzyme assays. (**f**) relative GS protein content of recombinant wheat GS isozyme crude extract. Upper panel shows TaGS immunoblot with same volumes of crude extract and lower panel shows relative content of recombinant TaGS protein in crude extract with same volume. Data represent means ± SE of at least three replicates.

To understand the catalytic properties of individual TaGS isoforms, the kinetic properties of recombinant TaGS isoforms expressed in *E. coli* were determined. The lysates of *E. coli* expressing recombinant TaGS protein were centrifuged and supernatants were used for GS enzyme assays. Kinetics of GS activities of recombinant TaGS1;1, TaGS1;2, TaGS1;3, and TaGS2 were plotted against the concentrations of Glu, hydroxylamine, and Gln in the reaction mixture (Figure 4).

TaGS1;2 activity was significantly inhibited when Glu was supplied at concentrations higher than 6 mM (Figure 4a) and was very weak with different concentrations of hydroxylamine when Glu was supplied at 50 mM (Figure 4b). However, TaGS1;2 activity was not inhibited when Glu was supplied at concentrations lower than 5 mM (Figure 4d), and was significantly increased with different concentrations of hydroxylamine when Glu was supplied at 5 mM (Figure 4e). With increasing Gln concentration, the activity of TaGS1;3 and TaGS2 remained stable. In contrast, in the reaction mixture with 60 mM Gln, the activity of TaGS1;1 and TaGS1;2 increased to about two and six times the activity in the reaction mixture without Gln, respectively (Figure 4c).

The specific activities plotted against the substrate concentrations showed saturation kinetics that follow the Michaelis–Menten equations, and the kinetic constants were calculated (Table 1). The four TaGS isoenzymes can be classified into groups by their affinity to substrates. TaGS1;1 (K_m_ = 0.65 ± 0.01 mM) and TaGS1;2 (K_m_ = 0.87 ± 0.01 mM) can be classified as isoenzymes with high affinity to Glu, while TaGS1;3 and TaGS2 exhibited low affinity to Glu (K_m_ = 4.13 ± 0.35 mM and 2.43 ± 0.27 mM, respectively). As for hydroxylamine, TaGS1;1 (K_m_ = 0.26 ± 0.02 mM) and TaGS2 (K_m_ = 0.36 ± 0.04 mM) showed higher substrate affinity than TaGS1;2 (K_m_ = 0.66 ± 0 mM) and TaGS1;3 (K_m_ = 0.64 ± 0.04 mM).

GS abundance in the supernatant was detected using Western blot. GS polypeptides were detected using polyclonal antibodies raised against TaGS1 and TaGS2. The relative content of GS was estimated by gray scanning using Image Lab analyzer software (Figure 4f). The results show that the ratio of TaGS1;1 to TaGS2 to TaGS1;2 to TaGS1;3 was 1:0.08:0.63:0.05. Based on the relative content of TaGS isoforms, we calculated the V_max_ of each one. The V_max_ of TaGS1;3 was the highest, about 15-fold, 4-fold, and 2-fold higher than that of TaGS1;1, TaGS1;2, and TaGS2, respectively (Table 1). 

Since individual TaGS isoforms have different catalytic properties, the metabolic status of carbon (C) and N in wheat will have different effects on their activity.

### 2.5. Effects of Nitrogen Nutrition on C/N Metabolite Status

In order to understand how individual TaGS isoforms participate in nitrogen assimilation, the C/N metabolism status of wheat under different N nutrition conditions was analyzed.

**Figure 5:**
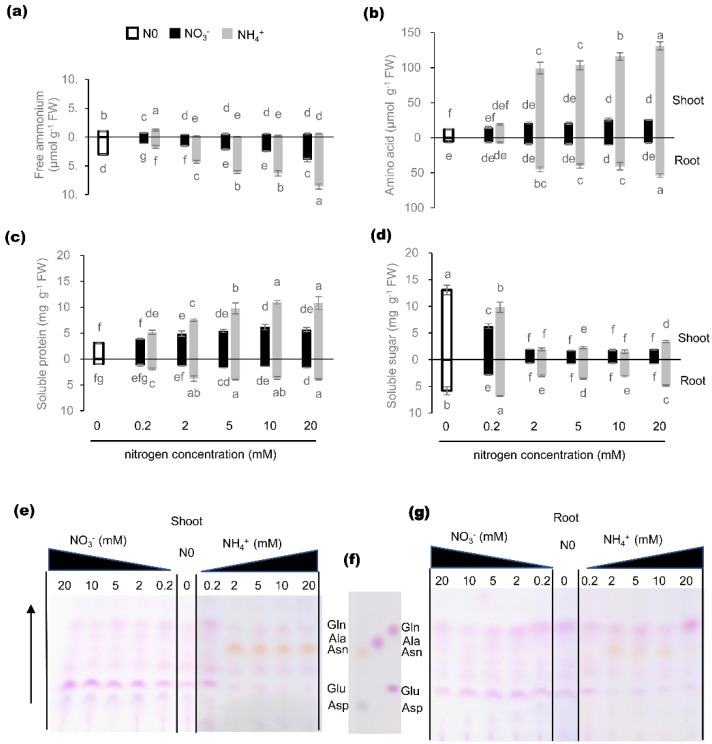
Carbon and nitrogen metabolite levels in response to different nitrogen regimes in shoot and root tissue. (**a**) ammonium, (**b**) free amino acid, (**c**) soluble protein, and (**d**) soluble sugar were determined. Individual amino acid components in response to different nitrogen regimes in shoot and root tissue. Amino acid components were analyzed with thin layer chromatography (TLC) and ninhydrin dye in (**e**) shoot and (**g**) root. (**f**) separation results of five amino acids (Gln, alanine (Ala), asparagine (Asn), Glu, and aspartate (Asp)) under the same conditions; 1.5 μg of free amino acid extracted from shoot and root tissues were loaded onto each lane of the TLC. Data are means of three independent biological replicates ± SD. Letters above samples indicate statistically significant differences where *p* < 0.05 according to one-way ANOVA Duncan post-hoc test.

Without N supply, shoot growth was significantly inhibited, while root growth was significantly promoted (Table S1). The free NH_4_^+^ produced by its own metabolic process was significantly higher in roots than in shoots (Figure 5a). In roots and shoots, free amino acid (Figure 5b), soluble protein (Figure 5c), and total nitrogen (Table S1) content were lower under nitrogen sufficiency, showing that nitrogen assimilation was inhibited under nitrogen deficiency.

Under 0.2 mM N^−^ supply, the content of free NH_4_^+^ in shoots was the highest (Figure 5a). With increasing N concentration, the content of free NH_4_^+^ in increased significantly in roots and decreased significantly in shoots and then kept stable, and the content in roots under NH_4_^+^ supply was higher than that under NO_3_^−^ supply. With increasing NO_3_^−^ supply, the content of NO_3_^−^ in shoots and roots increased synchronously (Figure S4) to about 10–60 times higher than that under relative NH_4_^+^ supply. These results indicate that NO_3_^−^ was main inorganic nitrogen for wheat seedlings to absorb and store, and roots can reduce the uptake NO_3_^−^ into NH_4_^+^ and can stand the high environmental NH_4_^+^ to some degree.

Under 0.2 mM N supply, the content of free amino acid in shoots and roots was the lowest (Figure 5b). With increasing NO_3_^−^ supply, the content of free amino acid in shoots and roots increased significantly and then kept stable. With increasing NH_4_^+^ supply, the content of free amino acid in shoots and roots increased significantly, and the content in shoots was about 2.5 times that in roots. Hence, the leaf is the major organ for nitrogen assimilation into amino acid and NH_4_^+^ promotes nitrogen assimilation in the leaf. 

Compared with the content of free amino acid, the content of soluble protein in shoots and roots was also the lowest under 0.2 mM N supply (Figure 5c). With increasing NO_3_^−^ supply, the content of soluble protein in shoots and roots increased and kept stable. With increasing NH_4_^+^ supply, the content of soluble protein in shoots increased significantly, while that in roots was just a little higher than that under 0.2 mM N supply. Moreover, the leaf soluble protein content under NH_4_^+^ supply was about 1.5–2 times higher than that under NO_3_^−^ supply, indicating that NH_4_^+^ can stimulate protein synthesis in the leaf while NO_3_^−^ ensures steady protein synthesis, for NO_3_^−^ can be mainly stored in leaf vacuoles (Figure S4).

Contrary to nitrogen assimilation, the content of soluble sugar in shoots and roots was the highest under 0.2 mM N supply (Figure 5d). With increasing N supply, the content of soluble sugar of wheat seedlings decreased significantly. Under NO_3_^−^ supply, the soluble sugar content in leaves was much higher than that in roots, which was the direct opposite to that under NH_4_^+^ supply. These results indicate that NH_4_^+^ can enhance soluble sugar transport from the leaf to the root, and further promote nitrogen assimilation in the root.

Nitrogen resources not only affected the content of free amino acid, but also changed the amino acid components observably (Figure 5e,g). Under NO_3_^−^ supply, the main components of amino acids in shoots were Glu and aspartate (Asp), and in roots were Glu and Gln. Under NH_4_^+^ supply, Asn was the main amino acid in shoots, and the main amino acids in roots were Gln and Asn. These results suggest that nitrogen resources may have distinct effects on nitrogen assimilation, transport, and storage.

## 3. Discussion

In plants, each GS isozyme plays a different role in nitrogen metabolism, and the expression of GS is strictly regulated at multiple levels [5,11,31,32]. GS proteins are responsible for the catalytic activity. However, previous studies about GS isoforms mainly focused on the mRNA level. In this study, using antibodies specific to individual TaGS isozymes, the expression differences of TaGS isoforms at the protein level were analyzed. Moreover, some new functions of TaGS isoforms were discovered by analyzing the effects of N supply on their expression and localization at the protein level, and their kinetic properties and nitrogen metabolism.

### 3.1. Nitrogen Nutrition Regulates Protein Accumulation of Individual TaGS Isozymes

Overexpressing GS1 gene in alfalfa significantly increased the *GS1* transcripts in the leaves, but did not change the level of GS1 polypeptides significantly [33]. In this study, the expression pattern of *TaGS1* at the mRNA level was not consistent with that at the protein level under different N treatments. NO_3_^−^ significantly promoted the accumulation of TaGS1;1 protein in the root (Figure 1b), and NH_4_^+^ inhibited the accumulation of TaGS1;2 protein in the root (Figure 1d). These results suggest that NO_3_^−^ and NH_4_^+^ may act as different exogenous signals to regulate the translation or post-translation of individual TaGS1 isoforms.

*TaGS2* transcript was in accordance with its protein level under different nitrogen treatments (Figure 1g,h). However, in transgenic tobacco with leaf-specific overexpression of *GS2*, the level of *GS2* transcript in the leaf was 15- to 18-fold higher than that in the wild type, but the GS2 protein was only about 2-fold higher in the transgenic plants than the wild type [35]. In our previous study, although there were many *TaGS2* transcripts in the roots of *TaGS2*-overexpressed tobacco, TaGS2 protein could not be detected [32]. These results show that TaGS2 should also have some regulatory mechanism at the protein level.

### 3.2. New Functions of TaGS Isozymes

**Figure 6:**
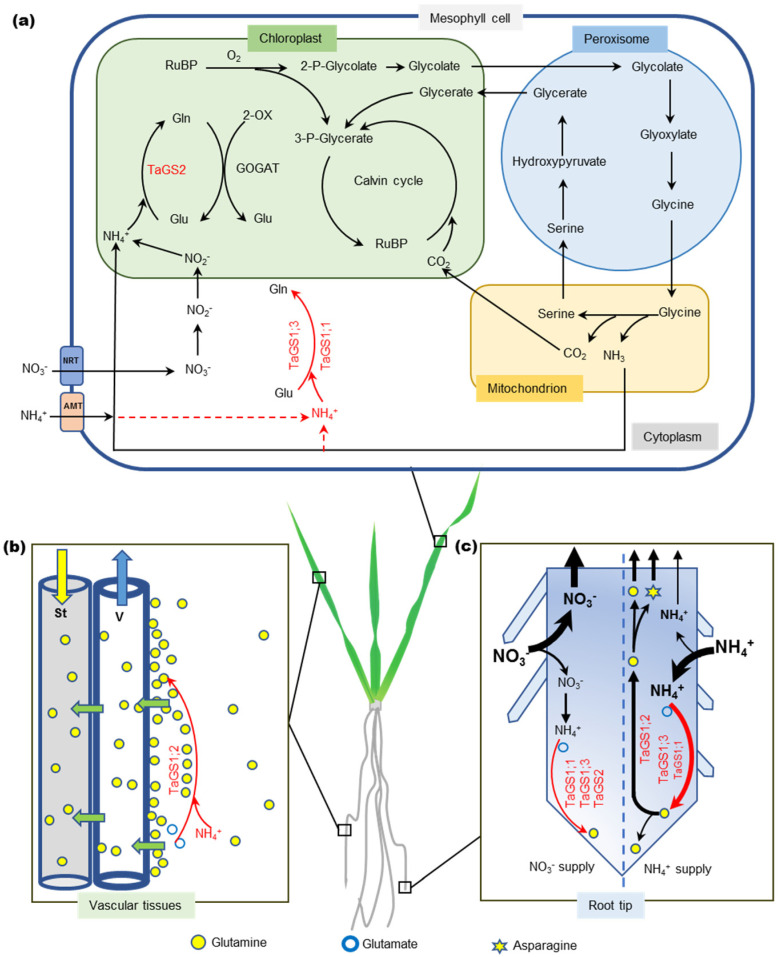
Schematic model of new functions of TaGS isoforms. (**a**) TaGS1;1 and TaGS1;3 were located in mesophyll and may participate in cytoplasmic assimilation of NH_4_^+^ released from photorespiration or absorbed by roots. (**b**) TaGS1;2 was located in vascular tissue of leaf and root and activated by Gln, hence responsible for nitrogen transport. (**c**) Under NO_3_^−^ supply (**left**), NO_3_^−^ stimulated TaGS1;1, TaGS1;3, and TaGS2 expression in root tip meristem and participated in assimilation of NH_4_^+^ from NO_3_^−^ reduction. Under NH_4_^+^ supply (**right**), TaGS1;3, with the highest V_max_, and TaGS1;1 were induced by NH_4_^+^ to express in root tip endodermis and may participate in relieving NH_4_^+^ toxicity. A large amount of TaGS1;2 was distributed in root tip vascular tissue, helping to transport Gln to shoots. NRT, nitrate transporter; AMT, ammonium transporter; St, sieve tube; V, vessel.

Previous studies showed that *TaGS1;1* transcript was present in perifascicular sheath cells [9], but we discovered that TaGS1;1 peptide was mainly localized in leaf mesophyll cells (Figure 2a,d,g). In the mesophyll cells, ammonium released from mitochondria during photorespiration is reassimilated in the chloroplast by GS2 [14]. Oliveira et al. [36] found that overexpression of cytosolic GS1 in leaf mesophyll cells could provide an alternate route to chloroplastic GS2 for the assimilation of photorespiratory ammonium. Hence, we speculate that TaGS1;1 in mesophyll cells participates in the reassimilation of cytoplasmic ammonium released from photorespiration or translocated from root absorption (Figure 6a).

A previous study showed that AtGln1;3, located in the pericycle of Arabidopsis roots, may be involved in xylem loading of Gln [21], but the mechanism is still unclear. Our interesting finding was that the activity of TaGS1;2 in the reaction mixture with 60 mM Gln was about six times that without Gln (Figure 4c). Gln, the product of GS, had no feedback inhibition to TaGS1;2, but significantly enhanced its activity in an opposite manner. TaGS1;2 had a high affinity for Glu (Table 1) and mainly localized around the xylem vessels (Figure 2g,i), hence we deduced that it catalyzed Gln synthesis in low Glu concentration, then the Gln returned to activate it resulting in subsequent Gln accumulation around vessels, which may facilitate the loading of Gln into xylem. Gln is the main translocation form of plant organic nitrogen [37]. In wheat, Gln concentration in leaf phloem sap was found to be dozens of times higher than in leaf tissue, where it is preferentially loaded into the vascular tissue for translocation [38], and Gln has amazing reverse concentration loading efficiency to vascular tissue [39]. Based on the above, we concluded that TaGS1;2 plays an important role in Gln loading into the vessels and transportation (Figure 6b). TaGS1;1 activity also increased with increased Gln concentration, but far less than TaGS1;2 activity (Figure 4c). Only when no N was supplied was TaGS1;1 found to surround vessels of xylem (Figure 2a). These results indicate that TaGS1;1 is also involved in loading Gln to the vessels, but has less importance than TaGS1;2. 

*TaGS1;3* is homologous to *HvGS1_3*, which is the only HvGS1 gene upregulated in the roots of barley grown under high NH_4_^+^, suggesting that it plays a role in the protection against NH_4_^+^ toxicity in roots [12]. The affinity of TaGS1;3 to Glu and hydroxylamine was the lowest of TaGS1, but it had the highest V_max_ (Table 1), suggesting that it has strong NH_4_^+^ assimilation ability. Under NH_4_^+^ supply, TaGS activity increased significantly in the roots (Figure 3), but subunits TaGS1;1 and TaGS1;2 did not show higher expression under NH_4_^+^ supply (Figure 1b,d). Located in pericycle cells of root endodermis of root tip and leaf mesophyll cells (Figure 2g,h), TaGS1;3 was significantly promoted by external NH_4_^+^ (Figure 2e,f), indicating that it mainly performs rapid NH_4_^+^ assimilation at high external NH_4_^+^ to protect the cells from the toxicity of high NH_4_^+^ concentration (Figure 6a,c). 

NO_3_^−^, as an important source of nitrogen absorbed by wheat roots, was reduced into NH_4_^+^ in leaves and roots, and then the NH_4_^+^ was assimilated by GS2 [12,20,40]. Previous studies showed that *GS2* transcripts were in roots [9,12,32]. We further discovered that TaGS2 in wheat roots was induced by NO_3_^−^ (Figure 1g), and located in the pericycle cells (Figure 2e) and the meristematic zone (Figure 2f) of roots only under NO_3_^−^ supply, which indicated it may participate in the assimilation of ammonia from NO_3_^−^ reduction in the root (Figure 6c).

### 3.3. TaGS Isozymes Synergistically Perform Nitrogen Assimilation and Translocation under Different N Nutrition Conditions

Without external nitrogen, shoot growth was inhibited while root elongation was promoted significantly in the early stage of wheat seedling (Table S1). N stress resulted in the accumulation of soluble sugar in leaves and roots (Figure 5d), and caused leaf senescence, proteolysis, and N remobilization [29]. TaGS1;1, located in the mesophyll cells (Figure 2a), assimilated ammonia released from the degradation of nitrogen-containing substances into Gln, and then Gln was loaded into the vessels by TaGS1;1 and TaGS1;2, which were distributed around the xylem vessels (Figure 2a). Xylem and phloem can exchange substances [41], so Gln loaded into the xylem vessels can also enter the phloem sieve tube and be transported to the root for its growth. TaGS1;1 and TaGS1;3, distributed in the meristematic zone of roots (Figure 2c), can participate in the reassimilation of NH_4_^+^ from metabolism. 

When NO_3_^−^ was supplied, it first translocated to the shoot through the xylem and reduced to NH_4_^+^ in the chloroplast of leaves, and then was assimilated into Gln by TaGS2. Some Gln remained in the leaves for leaf growth, some was loaded into xylem by TaGS1;2 and translocated to other parts through phloem. The more NO_3_^−^ was supplied, the more TaGS2 was induced in the leaves and roots (Figure 1) and the more NH_4_^+^ was located in the roots (Figure 5a), which may indicate that NO_3_^−^ reduction and assimilation occurred in both the leaves and roots. In roots, lots of TaGS1;1, TaGS1;3, and TaGS2 distributed in the meristematic zone (Figure 2f), while little was found in the maturation zone of roots, and TaGS1;2 was mainly distributed around the xylem vessels (Figure 2e); therefore, Gln was synthesized by TaGS1;1, TaGS1;3, and TaGS2 together, and then translocated to shoots via xylem by TaGS1;2. Most NO_3_^−^ is translocated to shoots and stored in vacuoles of mesophyll cells or directly stored in vacuoles of root cells [42], as a large amount of NO_3_^−^ was detected in shoots and roots (Figure S4). However, the free amino acid content was very low under NO_3_^−^ supply (Figure 5b), indicating that NO_3_^−^ was the major form for nitrogen storage.

NH_4_^+^ is an important inorganic nitrogen source, but high NH_4_^+^ concentration tends to be toxic to plants [43]. NH_4_^+^ penetrating into roots has to be immediately assimilated to Gln by GS to prevent toxicity [27]. Growing in an environment with high NH_4_^+^ concentration, plants will accumulate a large amount of ammonium [44] and maintain high levels of inorganic nitrogen assimilation in the roots to protect the photosynthetic parts of the plant against ammonium toxicity [45,46,47]. With increased NH_4_^+^ supply, NH_4_^+^ was largely accumulated in the roots (Figure 5a), resulting in the inhibition of root elongation (Table S1) but promotion of vascular tissue differentiation in the root tip (Figure 2i), which helped to translocate ammonium assimilate (Gln) from the root into the shoot in a timely manner. During this process, large amounts of TaGS1;1, TaGS1;2, and TaGS1;3 distributed in the root tips consumed the carbon skeleton translocated from the shoot for NH_4_^+^ assimilation, resulting in decreased soluble sugar content in the root (Figure 3b). A large amount of TaGS1;2 was distributed in the vascular tissue of root tips (Figure 2i), which helped to load Gln into the vascular tissue. Asparagine is another main compound for N storage and translocation due to its high N/C ratio and stability [48]. It is synthesized by asparagine synthase (AS) by the amidation of Asp using Gln as amino donor [48]. Under NH_4_^+^ supply, the root underwent high-intensity NH_4_^+^ assimilation, and Asn was accumulated in the shoot and root (Figure 5e,g), which suggests that Asn was synthesized in the root and then transported to the shoot.

When the external NH_4_^+^ exceeded the maximum amount stored and was assimilated by the roots, NH_4_^+^ may have been translocated to shoots through the xylem and assimilated in leaves. In leaves, TaGS1;1 and TaGS1;3 were distributed in the mesophyll cells (Figure 2g), and they may jointly participate in the assimilation of NH_4_^+^. Part of Gln can be loaded into the vascular tissue by TaGS1;2, which was distributed in the surrounding vessels of xylem and phloem companion cells (Figure 2g) and then translocated to the other tissue. 

Many studies have suggested that GS is closely related to crop nitrogen use efficiency [7,18,25,27,30,49], but the outcomes of single GS1 isozyme overexpression have generally been inconsistent [5]. We speculate that the main reason is that TaGS isoforms with no overlap role perform nitrogen assimilation and translocation synergistically under different N nutrition conditions. Therefore, if we want to improve crop NUE by overexpressing GS, the first step is to find out which GS isozyme is the limiting factor. As described by the cask effect, the amount of water in a cask is not determined by the highest block on the wall, but the shortest block.

## 4. Materials and Methods

### 4.1. Plant Growth Conditions and Experimental Design

For hydroponic treatments, uniform seeds were selected, surfaces were sterilized with 75% (*v*/*v*) ethanol for 1 min and rinsed with distilled water, and then the seeds were germinated in culture dishes covered with wet sterilized filter paper until the root length was about 1 cm. The uniform seedlings were transplanted to opaque containers and cultivated in distilled water. Hydroponic culture was carried out in a growth chamber with the following conditions: 22 °C ± 2 °C, 50% to 70% relative humidity, photon fluence rate of 300 μmol photons m^−2^·s^−1^, and 16 h light period. After 3 days, the seedlings were separated and grown on a modified Hoagland nutrient solution (Table S2, with NH_4_^+^ or NO_3_^−^ as the sole N source at concentrations of 0, 0.2, 2, 5, 10, and 20 mM. Each container contained 10 plants with 0.5 L nutrient, which was replaced every 3 d. After 12 days, shoots and roots were harvested individually and immediately frozen in liquid nitrogen, then stored at −80 °C for further experiments. In parallel, leaves and roots were selected and immediately immersed in fixative for immunolocalization studies. 

### 4.2. RNA Isolation and Quantitative Real-Time PCR

Total RNA was extracted from plant tissue using TRIzol Reagent (Thermo Scientific, Waltham, MA, USA). cDNA was synthesized using the Hiscript 1st Strand cDNA Synthesis Kit (Vazyme Biotech Co.,Ltd, Nanjing, China). Quantitative real-time PCR (qPCR) was performed on a Step One Real-Time PCR System (Life Technologies Corporation, Carlsbad, CA, USA) with AceQ qPCR SYBR Green Master Mix (Vazyme) for the assay. All primers (Sangon Biotech (Shanghai) Co., Ltd., Shanghai, China) used are shown in Table S3. The qPCR mix was composed of 10 µL AceQ qPCR SYBR Green Master Mix (Vazyme), 0.4 µL 50× ROX, 5 µL diluted cDNA 1:10 (*v*/*v*), 0.5 µL and 10 µM forward and reverse primers, respectively, and 3.6 µL of sterile nuclease-free water. Reactions proceeded according to the following program: 95 °C for 5 min, followed by 40 cycles of 95 °C for 15 s, 58 °C for 15 s, and 72 °C for 10 s. Fluorescence readings were taken during the elongation step (72 °C). Melting curves were obtained from 60 to 95 °C with a 1 °C increase every 10 s. Relative expression levels of genes were calculated using TaATPases (Ta54227) and TaTEF (Ta53964) genes [50] as internal control.

### 4.3. GS Activity Assay and Western Blotting

For the assay, 0.5 g of leaf or root sample was ground with liquid N_2_ and mixed with 1.5 mL Extraction Buffer (100 mM Tirs, 1 mM EDTA, 1 mM MgCl_2_, 1 mM phenylmethanesulfonyl fluoride, and 10 mM β-mercaptoethanol, pH 7.6). The extract was centrifuged at 13,000× *g* at 4 °C for 30 min. The supernatant was prepared for GS activity assay and Western blotting. Soluble protein content was determined by the Coomassie blue dye-binding method using bovine serum albumin as a standard.

Total GS activity was measured in accordance with a method described by Ma et al. [51]. One unit of GS activity consisted of the enzyme catalyzing the formation of 1 μmol of γ-glutamylhydroxamate/min at 25 °C, and total GS activity was determined by the μmol sum of γ-glutamylhydroxamate catalyzed by the whole enzyme per gram of fresh material in 1 h under the given conditions. Native polyacrylamide gel electrophoresis (PAGE) and in-gel GS activity staining were performed as previously described [30]. Western blotting was performed in accordance with a method described by Wei et al. [32]. For this procedure, 15 μg of soluble protein extracted from shoot or root was loaded onto each lane. Proteins were separated in 12.5% (*w*/*v*) polyacrylamide gel and electrophoretically transferred to 0.45 μm pore size PVDF membranes (Merck Millipore Ltd., Darmstadt, Germany) in transfer buffer (25 mM Tris-base and 192 mM Gly, 10% methanol) at 200 mA for 50 min. The membranes were blocked with TBST (20 mM Tris-base, 150 mM NaCl, and 0.05% (*v*/*v*) Tween 20, pH 7.4) containing 5% skim milk at 4 °C overnight. The membrane was incubated at 20 °C for 1.5 h with anti-GS polyclonal antibody. The dilution ratio of anti-TaGS1;1, anti-TaGS1;2, anti-TaGS1;3, and anti-TaGS2 antibody applied to the membrane was 1:30,000, 1:30,000, 1:10,000, and 1:10,000, respectively. After several washes with TBST, the membrane was incubated at room temperature for 1 h with horseradish peroxidase-conjugated goat anti-rabbit IgG (ABclonal Biotechnology Co., Ltd., Wuhan, Hubei, China) at 1:15,000. After several washes with TBST, the membrane was incubated at room temperature for 5 min using Clarity Western ECL reagent (Bio-Rad Laboratories, Lnc., Hercules, CA, USA ), and the signals were detected by Chemi DocTM XRS^+^ Imaging System (Bio-Rad). 

### 4.4. Immunolocalization Using Indirect Immunofluorescence Analysis

The tissues of leaf, root, and root tip were fixed in FAA fixative for at least 24 h. Their embedding in paraffin, sectioning, and immunofluorescence were performed by Servicebio (Wuhan Servicebio Technology Co., Ltd., Wuhan, Hubei, China). Anti-TaGS1;1, anti-TaGS1;2, anti-TaGS1;3, and anti-TaGS2 antibodies were diluted 1:200, 1:200, 1:500, and 1:50, respectively, in blocking solution. 

### 4.5. Expression of Recombinant Wheat GS Protein in E. coli

We used GS cDNA from wheat variety Yumai49 as the template and amplified the coding sequence (CDS) region for *TaGS1;1*, *TaGS1;2*, *TaGS1;3*, and *TaGS2* cDNA by PCR with the specific primers (Sangon) (Table S4). The PCR products were respectively cloned into the pET21a vector (Merck-Novagen, Darmstadt, Germany) and fully sequenced. The recombinant vectors were constructed using a ClonExpress One Step Cloning Kit (Vazyme). The CDSs of wheat GS were cloned into the *Nde* I and *Hind* Ⅲ sites of pET21a vector (Merck-Novagen). The recombinant vectors and empty pET21a vector were transformed into Rosetta (DE3) pLysS cell. Protein production was induced by the addition of isopropyl-b-d-thiogalactoside (IPTG) to a final concentration of 1 mM, with incubation in a shaker at 180 rpm. TaGS1;1, TaGS1;2, TaGS1;3, and TaGS2 were induced at 30 °C for 5 h, 12 °C for 17 h, 37 °C for 5 h, and 25 °C for 7 h, respectively, to get soluble protein. After induction, cells were harvested by centrifugation at 5000× *g* for 10 min at 4 °C. The pellet was suspended in breaking buffer (10 mmol/L Tris, 10 mmol/L MgCl_2_, 0.05% Triton X-100, 100 μg/mL PMSF, pH 7.5) and sonicated using a JY92-2D ultrasonic homogenizer (Ningbo Scientz Biotechnology Co. Ltd., Ningbo, China). The lysate was centrifuged at 12,000× *g* for 15 min at 4 °C and the supernatant was collected and used for kinetic measurements. Supernatant was kept on ice until it was used.

### 4.6. In Vitro Assay of Individual Recombinant Wheat GS Isozyme Activity

Determination of GS enzyme activity was based on an in vitro modified synthetase reaction, where the amount of produced γ-glutamyl monohydroxamate (GMH) is detectable by a stop reaction [51,52]. 

The crude extract of wheat GS protein recombinant *E. coli* was added to 800 µL reagent buffer. The reaction mixture was incubated at 25 °C for 15 min, terminated by adding 800 µL stop solution (123 mM FeCl_3_, 49 mM trichloroacetic acid, and 217 mM HCl) after centrifuging at 12,000× *g* for 5 min, and the absorbance of supernatant at 540 nm was determined. Reagent buffer always contained 40 mM magnesium sulfate and basically 100 mM imidazole, 50 mM ATP, 40 mM hydroxylamine, and 50 mM Na-glutamate, but concentrations varied depending on the actual kinetic assay (Na-glutamate: 0–120 mM; glutamine: 0–60 mM; and hydroxylamine: 0–80 mM). 

### 4.7. Metabolite Analysis

Amino acid, ammonium, and nitrate were determined according to Wei et al. [32]. Total N content was determined using a SEAL AutoAnalyzer 3 continuous flow analytical system (Bran + Luebbe, Hamburg, Germany), in accordance with the manufacturer’s instructions. Soluble sugar was determined using the anthrone colorimetric method [53]. Fine homogeneous powder of dry tissue (about 0.05 g) was extracted with 5 mL of 80% (*v*/*v*) ethanol at 80 °C for 20 min. The extract was centrifuged at 5000 rpm for 5 min, and the supernatant was transferred to a fresh 15 mL tube. The extraction was repeated 3 times. Then, 1 mL of supernatant was mixed with 4 mL of anthrone–H_2_SO_4_ reagent and heated at 80 °C for 10 min. Anthrone—H_2_SO_4_ reagent was mixed with 0.2% anthrone (*m*/*v*) added to 70% H_2_SO_4_ (*v*/*v*). After cooling, absorbance at 620 nm was determined and the soluble sugar content was calculated from the standard curve of sucrose.

The amino acid components were analyzed with thin layer chromatography. Amino acids were separated with phenol–water (3:1) as a developing solvent in silica gel G plate (10 cm by 20 cm). After developing solvent reached the top of the plate, the solvent front was marked with a pencil and then the solvent on the plate was vaporized in a 65 °C oven. The drying plate was taken out and sprayed with visualization reagent of 0.5% ninhydrin n-butyl alcohol, and then put into the oven at 65 °C and heated for 15 min to make the amino acids visible. Total free amino acids extracted from shoot and root tissues (1.5 μg) were loaded onto each lane of the TLC.

### 4.8. Statistics

One-way analysis of variance with a Duncan post hoc test was performed using SPSS version 13.0 (IBM, Chicago, IL, USA).

## Figures and Tables

**Table 1:** Kinetic properties of wheat GS isoenzymes.

Name	Km * (mM)		Vmax ** (nKat/1 Unit Protein)	
	Glu	Hydroxylamine	Glu	Hydroxylamine
TaGS1;1	0.65 ± 0.01 d	0.26 ± 0.02 c	0.13 ± 0.001 d	0.1 ± 0.002 d
TaGS1;2	0.87 ± 0.01 c	0.66 ± 0.003 a	0.5 ± 0.002 c	0.36 ± 0.003 c
TaGS1;3	4.13 ± 0.35 a	0.64 ± 0.04 a	1.95 ± 0.07 a	1.63 ± 0.08 a
TaGS2	2.43 ± 0.27 b	0.36 ± 0.04 b	0.92 ± 0.04 b	1.00 ± 0.04 b

* For TaGS1;1, TaGS1;2, TaGS1;3, and TaGS2, the concentration of Glu used for curve fitting was 0–40 mM, 0–5 mM, 0–120 mM, and 0–120 mM, and the concentration of hydroxylamine was 0–80 mM, 0–50 mM, 0–30 mM, and 0–80 mM, respectively. The volume of individual recombinant wheat GS isozyme crude extract with 200 μL of TaGS1;1, 150 μL of TaGS1;2, 450 μL of TaGS1;3, and 300 μL TaGS2 was used for GS enzyme assays. ** One kat of enzyme activity was defined as 1 mol γ-glutamyl monohydroxamate (GMH) synthesized per second at 25 °C. Data are means of three independent biological replicates ± SD. Letters above samples indicate statistically significant differences where *p* < 0.05 according to one-way ANOVA Duncan post-hoc test.

## Supplementary Materials

Supplementary materials can be found at https://www.mdpi.com/1422-0067/21/17/6299/s1.

## Abbreviations

GS	Glutamine synthetase
N	Nitrogen
NH_4_^+^	Ammonium
NO_3_^−^	Nitrate
Gln	Glutamine
Asn	Asparagine
Glu	Glutamate
Asp	Aspartate
Ala	Alanine
AS	Asparagine synthase
NUE	Nitrogen use efficiency
CDS	Coding sequence
GMH	γ-glutamyl monohydroxamate
IPTG	Isopropyl-b-d-thiogalactoside
TLC	Thin layer chromatography
