# Supplementary material for: Nitrogen Regulating the Expression and Localization of Four Glutamine Synthetase Isoforms in Wheat (Triticum aestivum L.)
Source: Int J Mol Sci. 2020 Aug 31;21(17):6299. doi: 10.3390/ijms21176299 (PMC7504200; doi:10.3390/ijms21176299)
Supplement: Supplementary file 1 [file ijms-21-06299-s001.pdf]

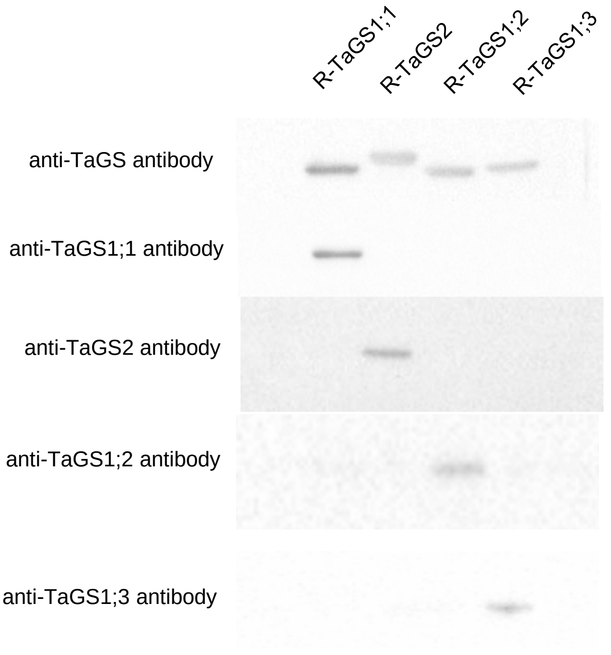

Figure S1 The specificity of anti-GS antibodies to the individual recombinant TaGS subunits (R-TaGS). The lysates of E. coli expressing recombinant TaGS protein were centrifuged and supernatants were used for western blot assays. The supernatants volumes of R-TaGS1;1, R-TaGS2, R-TaGS1;2, and TaGS1;3 loaded are 1μL, 7 μL, 0.85 μL, and 8.5 μL respectively. The dilution ratio of the anti-TaGS, anti-TaGS1;1, anti-TaGS2 anti-TaGS1;2 and anti-TaGS1;3 antibody is 1:5000, 1:30000, 1:10000, 1:30000 and 1:10000, respectively.

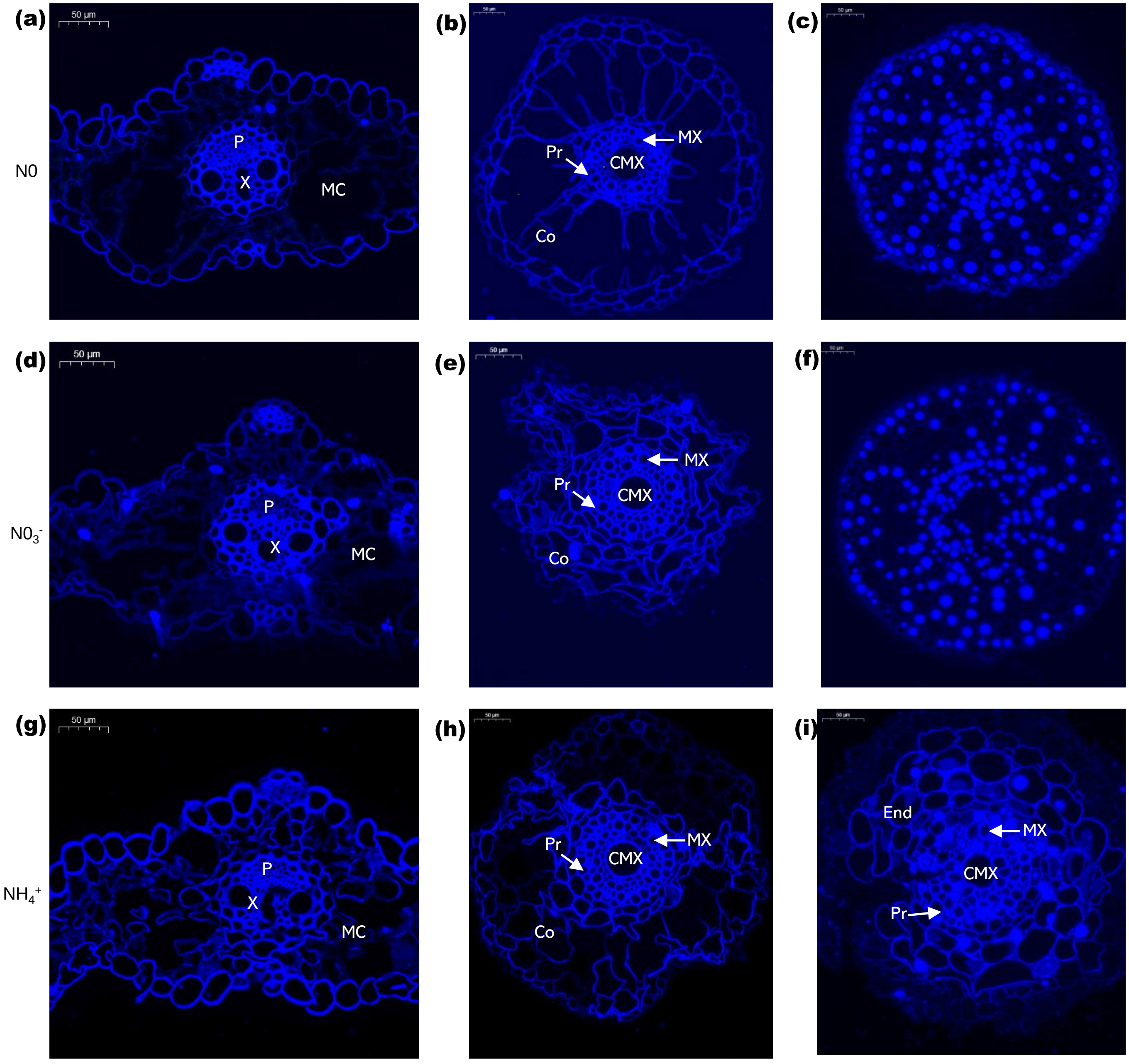

Figure S2 Histological structural observations of wheat tissue under different nitrogen regimes. Three-day-old seedlings were separated and grown on a modified Hoagland nutrient solution for 12 days, without N supply (N0), with 5mM NO_3^-or 5mM NH_4^+ as the sole N source, and then wheat materials were prepared for section. The tissues of transverse section including the leaf (**a, d, g**), the maturation zone (**b, e, h**) and meristematic zone (**c, f, i**) of root. DAPI glowed blue by UV excitation wavelength 330-380 nm and emission wavelength 420 nm. MX, metaxylem; P, phloem; X, xylem; VB, vascular bundle; CMX, central metaxylem; End, endodermis; Pr, pericycle; Co, cortex; MC, mesophyll cells.

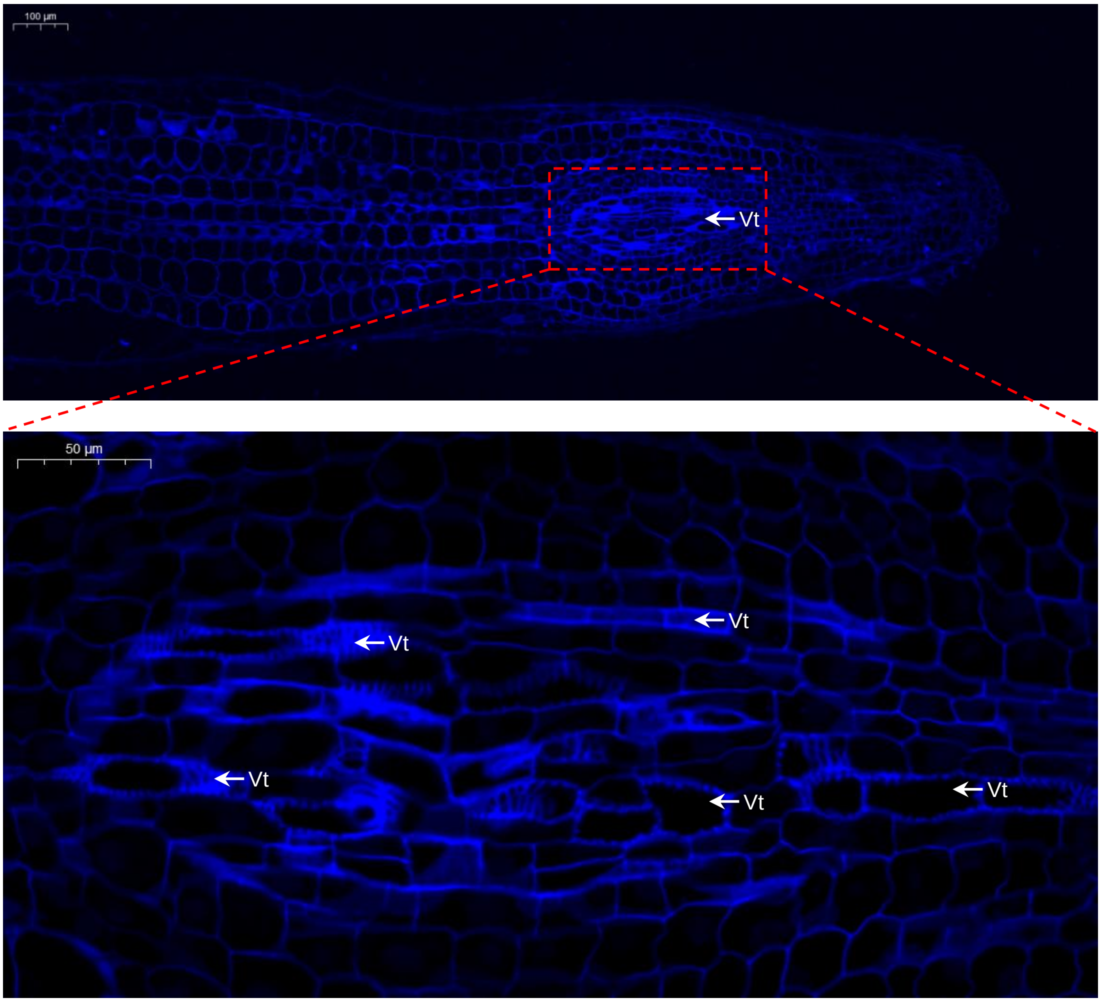

Figure S3 Observation of longitudinal root tip structure of wheat seeding growing for 12 days under 5 mM NH_4^+ as the sole N source. DAPI glowed blue by UV excitation wavelength 330-380 nm and emission wavelength 420 nm; Vt, vascular tissue.

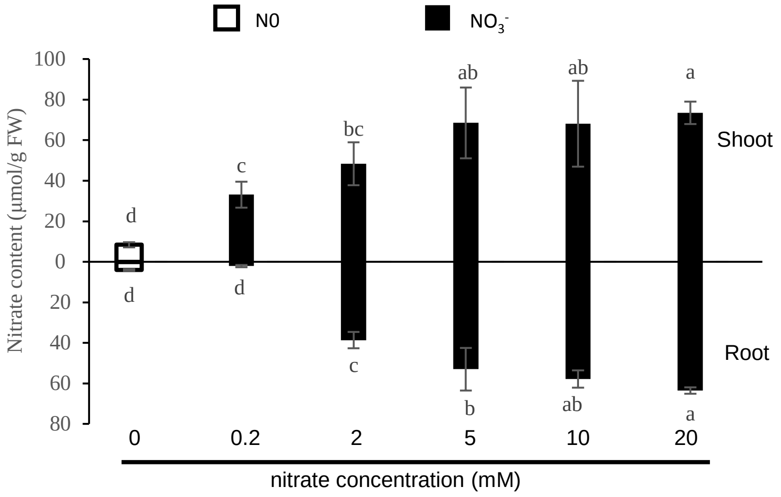

Figure S4 Effect of NO_3^- supply on the content of NO_3^- in the shoots and roots. Data are means of three independent biological replicates ± SD. Letters above samples indicate statistically significant differences where $P < 0.05$ according to one-way ANOVA Duncan post-hoc test.

Table S1 Effect of nitrogen regimes on dry weight, fresh weight, root length, and nitrogen content.

			N0	0.2mM	2mM	5mM	10mM	20mM
Dry weight (mg/plant)	Shoot	NO_3^-	34.7±0.6 j	44.3±1.9 i	67.3±2.8 b	66.8±1.3 bc	64.9±2.5 bc	74±3.2 a
		NH_4^+		48.1±1.1 hi	58.3±4.3 def	62.1±1.7 cd	57±2.9 ef	56.6±3 ef
	Root	NO_3^-	36±2.8 a	37.6±1.5 a	26.8±2.2 cd	24.9±1.3 d	25.2±1.3 d	25±2.2 d
		NH_4^+		33.6±1 b	14.7±1.8 ef	17.8±1.2 e	16.9±0.2 ef	16.6±0.5 ef
Fresh (mg/plant)	Shoot	NO_3^-	248.8±12.6 k	382.4±16.1 fi	763.4±67 a	706.6±22.6 b	680.3±38.6 b	757.3±18.2 a
		NH_4^+		366.7±16.7 ij	441.1±16.4 de	465.6±8.4 cd	423.3±20.3 de	408.9±15.4 ef
	Root	NO_3^-	375.3±41.5 bc	448.4±20.8 a	398.2±68.9 b	353.3±7.5 bc	368.6±25.7 bc	368.3±46.4 bc
		NH_4^+		350±5.8 c	133.3±3.3 d	145.6±11.7 d	152.2±15 d	142.2±5.1 d
Root length (cm)		NO_3^-	52.6±3.3 a	43.8±3.8 c	35.5±2.4 de	36.4±3.9 d	34.1±2.9 de	29.3±3.4 f
		NH_4^+		35.5±3.1 de	17.7±1.7 g	18.1±1.9 g	16.5±1.4 g	18.2±1.7 g
Nitrogen content (mg N /Plant)	Shoot	NO_3^-	0.6±0.03 g	1.03±0.08 f	3.08±0.23 b	2.85±0.16 bc	2.65±0.39 cde	3.62±0.14 a
		NH_4^+		0.82±0.03 fg	2.59±0.22 cde	2.76±0.19 bcd	2.48±0.25 de	2.38±0.09 e
	Root	NO_3^-	0.42±0.02 ef	0.62±0.05 b	0.85±0.12 a	0.81±0.03 a	0.84±0.02 a	0.82±0.1 a
		NH_4^+		0.51±0.02 cde	0.47±0.06 de	0.63±0.05 b	0.59±0.03 bc	0.54±0.01 bcd

Note: Data are means of three independent biological replicates ± SD. The different letters above each sample indicate statistically significant differences where $P < 0.05$ according to one-way ANOVA Duncan post-hoc test.

Table S2 Composition of nutrient solution treated with different nitrogen sources

	KH_2PO_4,	$MgSO_4$	KCl	$CaCl_2$	$Ca(NO_3^-)_2$	NH_4Cl
N0	0.2mM	1mM	1.5mM	2.5mM	0	0
0.2mM NO_3^-	0.2mM	1mM	1.5mM	2.4mM	0.1mM	0
2mM NO_3^-	0.2mM	1mM	1.5mM	1.5mM	1mM	0
5mM NO_3^-	0.2mM	1mM	1.5mM	0	2.5mM	0
10mM NO_3^-	0.2mM	1mM	1.5mM	0	5mM	0
20mM NO_3^-	0.2mM	1mM	1.5mM	0	10mM	0
0.2mM NH_4^+	0.2mM	1mM	1.5mM	2.5mM	0	0.2mM
2mM NH_4^+	0.2mM	1mM	1.5mM	2.5mM	0	2mM
5mM NH_4^+	0.2mM	1mM	1.5mM	2.5mM	0	5mM
10mM NH_4^+	0.2mM	1mM	1.5mM	2.5mM	0	10mM
20mM NH_4^+	0.2mM	1mM	1.5mM	2.5mM	0	20mM

Note: Nutrient solutions treated with different nitrogen sources had the same content of trace elements (20 µM Fe-EDTA, 6.7 µM $MnSO_4$, 0.32 µM $CuSO_4$, 0.77 µM $ZnSO_4$, 46 µM H_3BO_3, 0.5 µM H_2MoO_4, 0.2 µM $CoCl_2$, 5µ M KI). The pH value of the nutrient solution was adjusted to 6.0 with HCl or NaOH.

Table S3 List of primers used for qPCR.

Gene Name	Primer	Sequence(5'-3')
TaGS1;1	*TaGS1;1-F*	AAGGACGGCGGGTTC AA
	TaGS1;1-R	GCGATGTGCTCCTTGTGCTT
TaGS1;2	*TaGS1;2-F*	GACAACTTCCTTGTTATGTGCCAC
	TaGS1;2-R	TGTGCCTCTTGTTCGTGGG
TaGS1;3	*TaGS1;3-F*	CTG TGA CTG CTA TGC GCC TAA C
	TaGS1;3-R	CCG CGT TGT ACC GCT TGT
TaGS2	*TaGS2-F*	GGT TGA CAG GGC TAC ACG AGA
	TaGS2-R	GAG CAG CCA CGG TTC GC
ATPase	*ATPase-S*	ATACGCCATCAGGGAGAACATC
	ATPase-A	AGGGTTGTCCTTCCTCCGC
TaTEF1	*TaTEF1-S*	GGTTGTGGAGACCTTTGCTACTTAC
	TaTEF1-A	AACAGCCACAGTTTGCCTCAT

Table S4 Primers used to amplify coding sequence (CDS) of TaGS1;1, TaGS1;2, TaGS1;3, and TaGS2 from wheat.

Gene Name	Primer	Sequence(5'-3')
TaGS1;1	*TaGS1;1-F*	ACCCGCCTTCCTTCCTGC
	TaGS1;1-R	CGATGATGCGACCTACCTAAGC
TaGS1;2	*TaGS1;2-F*	CATTCCCTCCTTGCGAG
	TaGS1;2-R	AAATGGAAACACGAAACG
TaGS1;3	*TaGS1;3-F*	GAAGAAGAAGAAGAGGTAGCCATG
	TaGS1;3-R	AACAGAACCCATCAAAGCCAC
TaGS2	*TaGS2-F*	GCGGAGTAAGTAAGTAAGCAGC
	TaGS2-R	CATGCGGAGCGGTTCTAC
